# Mesenchymal Stem Cells and Extracellular Vesicles: Bridging the Translational Gap in Regenerative Medicine

**DOI:** 10.3390/ijms27125423

**Published:** 2026-06-16

**Authors:** Ramya Lakshmi Rajendran, Prakash Gangadaran

**Affiliations:** 1 Department of Nuclear Medicine, School of Medicine, Kyungpook National University, Daegu 41944, Republic of Korea; ramyag@knu.ac.kr; 2 Cardiovascular Research Institute, Kyungpook National University, Daegu 41944, Republic of Korea; 3 BK21 FOUR KNU Convergence Educational Program of Biomedical Sciences for Creative Future Talents, Department of Biomedical Sciences, School of Medicine, Kyungpook National University, Daegu 41944, Republic of Korea

## 1. Opening Perspective: A Field at Two Different Stages

The field of regenerative medicine has reached a significant milestone, evolving from the established transplantation of mesenchymal stem cells (MSCs) to cell-free therapies using MSC-derived extracellular vesicles (EVs). Although MSC-derived EVs remain in the early stages of development, the recent approval of Ryoncil by the Food and Drug Administration (FDA) highlights the potential of whole-cell transplantation as a breakthrough in regenerative medicine. To fully realize this translational potential, regulatory challenges must be overcome and rigorous potency testing conducted despite the greater stability and lower immunogenicity of EVs. By addressing these obstacles, we aim to shift the paradigm, transforming these “tiny giants” from laboratory tools into programmable nanomedicines for widespread clinical application.

## 2. Mesenchymal Stem Cells in the Clinic: Progress with Persistent Limitations

MSCs demonstrate clinical benefits in treating conditions such as Graft-versus-Host Disease (GVHD), COVID-19, and epidermolysis bullosa (EB), owing to their ability to modulate the immune system [1,2,3]. In GVHD, MSCs exert immunosuppressive and immunoregulatory effects that contribute to immune modulation. They achieve this by secreting proteins, peptides, and functional RNA, and other factors, including those delivered via EVs [1]. During the COVID-19 pandemic, over 70 clinical trials investigated MSCs derived from umbilical cord, bone marrow, and adipose tissue as treatment agents. Preliminary data suggest some benefits, primarily through suppression of the COVID-19 cytokine storm, but further investigation is required [2]. In EB, MSCs show safety and potential anti-inflammatory effects by suppressing Monocyte Chemoattractant Protein-1 and soluble CD40 Ligand [3]. However, due to MSC heterogeneity, which contributes to inconsistent outcomes in some of the further clinical trials, universal translation remains under review [4]. The field is now shifting toward validated potency assays, marker-guided patient screening, and engineered delivery vehicles to improve cell engraftment and address these persistent challenges, thereby enhancing clinical outcomes.

## 3. Extracellular Vesicles as the Functional Extension of Mesenchymal Stem Cell Therapy

The therapeutic paradigm of MSCs has shifted from cell replacement to a primarily paracrine-mediated regenerative strategy. Initially, MSCs were considered living drugs capable of engrafting into damaged tissues and differentiating into functional cell types. However, growing evidence suggests that the regenerative effects of MSCs are predominantly mediated by their secretome, particularly EVs, which are key mediators of intercellular communication [5]. These nanosized lipid bilayer vesicles, including exosomes and microvesicles, carry diverse cargoes of proteins, lipids, messenger RNAs, microRNAs, and long noncoding RNAs that modulate the phenotype and function of recipient cells. Through this cargo transfer, EVs coordinate multiple regenerative pathways involved in tissue repair, inflammation control, and angiogenesis, thereby transforming regenerative medicine from a cell-based framework to a more targeted acellular therapeutic strategy [6].

MSC-derived EVs are increasingly recognized as functional extensions of stem cell therapy, exhibiting biological activities similar to those of MSCs. Often described as naturally occurring endogenous nanocarriers [7], they retain the regenerative capacity of MSCs while avoiding many limitations of direct stem cell transplantation. Among the various MSC types studiedsuch as bone marrow-derived MSCs, adipose-derived stem cells, and human umbilical cord-derived MSCs (hucMSCs).HucMSCs are particularly promising due to their high proliferative capacity, superior differentiation potential, and fewer ethical concerns [8,9,10,11]. MSC-derived EVs can influence cell behavior in injured tissues and promote regenerative responses by enhancing intercellular communication without requiring live transplanted cells. EV-based therapies offer several translational and therapeutic advantages over conventional MSC-based treatments. As a cell-free therapeutic approach, EVs circumvent many safety concerns associated with live cell injections, including uncontrolled differentiation, arterial obstruction, pulmonary embolism, and ectopic tissue integration [6]. Moreover, EVs are less immunogenic because they lack a nucleus and express low levels of major histocompatibility complex molecules, minimizing the risk of immune rejection and malignancy. Owing to their nanoscale size, EVs can more effectively penetrate biological barriers and access tissues [6]. Pharmacologically, EVs demonstrate enhanced stability and handling properties, which facilitate preservation, transport, and long-term storage through cryopreservation or lyophilization. These attributes make MSC-derived EVs promising, safer, and more controllable biopharmaceuticals for regenerative therapy and clinical translation [12].

MSC-derived EVs show significant regenerative potential in preclinical models of burns, diabetic wounds, ischemic injury, and radiation-induced tissue damage. These vesicles enhance fibroblast proliferation, keratinocyte migration, collagen deposition, and reepithelialization, thereby facilitating tissue remodeling and wound healing [13]. Beyond tissue repair, EVs exhibit potent immunomodulatory properties, including modulation of macrophage polarization and suppression of inflammatory cytokine pathways, such as the miR-181c signaling pathway. These anti-inflammatory effects are crucial for chronic wounds associated with persistent inflammation [14]. Moreover, MSC-derived EVs exhibit significant pro-angiogenic properties by transferring angiogenic microRNAs and growth factors. These molecules activate signaling pathways such as ERK1/2 and PI3K/Akt/mTOR, promoting neovascularization and restoring blood flow in injured tissues. However, despite promising mechanistic and preclinical findings, the clinical translation of MSC-derived EV-based therapeutics remains limited [15]. The primary challenges for the clinical application of EVs include variability in EV preparations, the absence of standardized isolation and characterization protocols, diversity in donor cell sources, and the lack of established dosing guidelines. Additionally, EVs are often rapidly cleared from the body, which limits their therapeutic potential [16,17]. Exosome technologies continue to be enhanced through strategies such as donor cell preconditioning, EV cargo engineering, and integration with biomaterial-based delivery systems, including hydrogels, to overcome existing limitations, enhance stability, and enable targeted release. Scalable manufacturing processes and standardized characterization methods are essential for clinical translation and reproducibility. Collectively, these advances position MSC-derived EVs as promising therapeutic platforms [18].

## 4. Why Extracellular Vesicles Are Not Yet in Routine Clinical Use

MSC-derived EVs demonstrate significant regenerative and immunomodulatory potential in preclinical studies but have not been adopted into routine clinical practice. This gap between strong biological efficacy and limited clinical adoption remains a major translational challenge in regenerative medicine [19]. The primary obstacles no longer lie in EV biology itself but in standardization, production, regulatory classification, and clinical validation. Despite rapid advances in EV research, relatively few EV-based systems have reached clinical application, highlighting the need for methodological and infrastructural improvements [20,21]. Standardized isolation and characterization techniques remain lacking, representing a major barrier to clinical translation. Researchers currently employ various isolation methods, including ultracentrifugation, precipitation-based approaches, size-exclusion chromatography, and affinity-based techniques, each producing EV populations with differing purity and molecular composition [22]. Ultracentrifugation is the experimental “gold standard” for EV isolation; however, it is labor-intensive, equipment-dependent, and difficult to scale for clinical manufacturing. In contrast, precipitation-based procedures produce more particles but often co-isolate impurities such as proteins and lipoproteins, reducing EV preparation quality [23]. Furthermore, variability in isolation methods can selectively enrich distinct EV subpopulations, resulting in differences in treatment potency and poor reproducibility across trials. Collectively, these challenges hinder the development of reliable, clinically standardized EV therapies [24].

The translational challenges of EV therapies are further complicated due to inconsistencies in EV characterization and manufacturing. Although the MISEV 2018 and 2023 guidelines recommend standardized characterization methods—including imaging, nanoparticle tracking analysis, and protein marker profiling—many preclinical studies still lack adequate EV validation [25,26]. Commonly used purity metrics, such as the particle-to-protein ratio, may not accurately reflect EV composition because impurities, including lipoproteins and protein aggregates, can be co-isolated. This issue is particularly significant in plasma-derived EV studies, where lipoproteins outnumber EVs and share similar physical characteristics [27]. Moreover, the absence of membrane-protection assays using RNase, DNase, and protease treatments prevents the determination of whether identified biomarkers are truly encapsulated within EVs or represent external contaminants. Consequently, no universally accepted definition of a pure, physiologically active EV preparation has been established [25,26]. Manufacturing challenges further impede clinical translation because current isolation technologies are difficult to scale while maintaining reproducibility and Good Manufacturing Practice (GMP) compliance. Variations in centrifugation parameters, membrane systems, and purification protocols can significantly influence EV yield and therapeutic potency, resulting in batch-to-batch variability. Emerging technologies such as tangential flow filtration, ligand-based exosome affinity purification, and anion-exchange chromatography show promise for scalable production; however, additional purification is generally required to achieve clinical-grade purity [28,29]. Furthermore, undefined active pharmaceutical ingredients and mechanisms of action complicate the development of standardized production pipelines. Regulatory uncertainty is another barrier to widespread clinical implementation. EV therapies do not fit neatly within existing regulatory frameworks for biologics or pharmaceuticals, creating an approval gray area [30]. Limited understanding of EV pharmacokinetics, biodistribution, long-term safety, and repeated-dose administration further complicates regulatory evaluation. Overly rigorous purification methods may remove surface-associated biomolecules known as the “EV corona,” thereby reducing therapeutic efficacy. Collectively, these challenges suggest that the primary barrier to EV translation is not their biological potential but the lack of a robust translational infrastructure, including standardized production, characterization, and regulatory frameworks [31,32].

MSC-derived EVs show promising immunomodulatory and regenerative properties in preclinical studies [33]. However, successful translation of MSC-derived EVs from bench to bedside requires standardized characterization, scalable GMP-compliant manufacturing, harmonized regulatory frameworks, validated potency assays, and well-designed clinical trials [34]. Until these limitations are addressed, EV therapies will remain scientifically promising but clinically underutilized. Nevertheless, advances in engineered exosomes, scalable purification technologies, and related initiatives are bringing the field closer to safe, clinically viable cell-free regenerative therapies [35].

## 5. Lessons from Mesenchymal Stem Cell Clinical Translation

MSCs derived from bone marrow, adipose tissue, and umbilical cord exhibit variable therapeutic efficacy [36,37,38]. Their efficacy also varies across disease contexts, such as GVHD, Crohn’s disease, osteoarthritis, acute respiratory distress syndrome, and cardiac repair [1,39,40,41,42]. MSC clinical translation established a significant framework for therapeutic development, highlighting the importance of standardized closed-system platforms and cGMP-compliant manufacturing to reduce product variability [43]. The field must implement validated potency assays, such as measuring IDO activity or VEGF secretion, that accurately reflect the unique mechanism of action of a product. This lesson, drawn from previous trial failures, highlights that standard phenotypic markers (e.g., International Society for Cellular Therapy criteria) are insufficient predictors of therapeutic success [44]. Furthermore, ensuring consistent efficacy across diverse pathologies requires a shift from a one-size-fits-all model to individualized approaches based on patient stratification and biomarker-guided selection. Researchers can significantly improve the translation of MSC-derived EVs into reliable, standardized clinical therapies by combining strict manufacturing standards, advanced delivery systems such as hydrogels, and functional characterization frameworks [45]. However, clinical translation remains challenging due to MSC heterogeneity, manufacturing inconsistencies, and the limited reproducibility of animal model findings in human patients [46,47,48]. Stringent regulatory requirements from agencies such as the U.S. FDA and the European Medicines Agency also require standardized production processes and clearly defined product attributes for successful commercialization [49,50].

## 6. Clinical Opportunities: Where Extracellular Vesicles Can Lead Next

EVs can be engineered for targeted drug delivery through surface modifications, such as hyaluronic acid and folic acid, to improve cellular uptake at specific disease sites [51]. To mitigate the risk of uncontrolled differentiation or tumorigenesis associated with live MSC administration in patients with chronic inflammatory diseases such as asthma or autoimmune disorders, EV-based therapies offer a safer option for repeated dosing. Furthermore, EVs provide greater control over therapeutic cargo, enabling the loading of specific microRNAs or anti-inflammatory cytokines, particularly interleukin-10, to promote vascular regeneration and facilitate the M1-to-M2 macrophage phenotypic switch essential for tissue repair [52]. EVs represent a scalable, low-immunogenicity platform for treating complex diseases, from pulmonary fibrosis to chronic wounds, while maximizing local therapeutic concentrations and minimizing systemic side effects.

## 7. Insights from This Special Issue

The contributions in this Special Issue on “Applications of Stem Cells and Extracellular Vesicles in Regenerative Medicine” reinforce a central thesis: MSCs and EVs represent successive stages of the same translational continuum rather than competing therapeutic modalities. Over two decades of MSC research have generated clinical experience across diverse applications, including immune regulation in GVHD, musculoskeletal repair, and wound healing. This body of research has done more than produce a few approved therapies; it has rigorously established key requirements for successful biological therapies, such as GMP-compliant manufacturing, potency-aligned release criteria, patient stratification, and meaningful clinical endpoints. EV-based therapies are now building on this hard-won framework. As demonstrated in this Special Issue, MSC-derived EVs retain the regenerative and immunomodulatory functions of their parent cells while mitigating the risks associated with viable cell administration. This advantage enables safer repeat dosing, precise cargo engineering, and scalable bioreactor-based production. This trajectory is evident across applications ranging from neurodegenerative diseases and chronic inflammation to precision oncology and ischemic repair. The field is evolving from cell transplantation to programmable nanomedicine, with MSC clinical translation providing proof of concept and a regulatory framework. The work presented here provides a foundation for the next generation of EV-based therapies.

## Data Availability

No new datasets were generated or analyzed in this study. All data discussed are derived from previously published articles cited in the reference list.

## References

[1] Kelly K., Rasko J.E.J. (2021). Mesenchymal Stromal Cells for the Treatment of Graft Versus Host Disease. Front. Immunol..

[2] Chen L., Qu J., Kalyani F.S., Zhang Q., Fan L., Fang Y., Li Y., Xiang C. (2022). Mesenchymal Stem Cell-Based Treatments for COVID-19: Status and Future Perspectives for Clinical Applications. Cell. Mol. Life Sci..

[3] Maseda R., Arriba M.C., Martínez-Santamaría L., Jiménez E., Herráiz-Gil S., Illera N., Quintana-Castanedo L., García M., Suárez-Sancho S., Yáñez R. (2026). MesenSistem-EB: Systemic Haploidentical Mesenchymal Stem Cell Therapy in Recessive Dystrophic Epidermolysis Bullosa Associated with Clinical Benefits and Correlated with MCP1 and sCD40L Dynamics. Front. Immunol..

[4] Lowenstein P.R., Castro M.G. (2009). Uncertainty in the Translation of Preclinical Experiments to Clinical Trials. Why Do Most Phase III Clinical Trials Fail?. Curr. Gene Ther..

[5] Trigo C.M., Rodrigues J.S., Camões S.P., Solá S., Miranda J.P. (2025). Mesenchymal Stem Cell Secretome for Regenerative Medicine: Where Do We Stand?. J. Adv. Res..

[6] Shimizu Y., Inoue Y., Matsuura N., Ishii T., Sowa Y., Sunami H., Ntege E.H. (2026). Mesenchymal Stromal Cell-Derived Extracellular Vesicles in Regenerative Medicine: Standardisation, Bioengineering and Clinical Translation. Regen. Ther..

[7] Li S., Zhang J., Sun L., Yang Z., Liu X., Liu J., Liu X. (2025). Mesenchymal Stem Cell-Derived Extracellular Vesicles: Current Advances in Preparation and Therapeutic Applications for Neurological Disorders. Front. Cell Dev. Biol..

[8] Yea J.-H., Kim Y., Jo C.H. (2023). Comparison of Mesenchymal Stem Cells from Bone Marrow, Umbilical Cord Blood, and Umbilical Cord Tissue in Regeneration of a Full-Thickness Tendon Defect In Vitro and In Vivo. Biochem. Biophys. Rep..

[9] Kim J.-H., Jo C.H., Kim H.-R., Hwang Y. (2018). Comparison of Immunological Characteristics of Mesenchymal Stem Cells from the Periodontal Ligament, Umbilical Cord, and Adipose Tissue. Stem Cells Int..

[10] Nagamura-Inoue T., He H. (2014). Umbilical Cord-Derived Mesenchymal Stem Cells: Their Advantages and Potential Clinical Utility. World J. Stem Cells.

[11] Russo E., Caprnda M., Kruzliak P., Conaldi P.G., Borlongan C.V., La Rocca G. (2022). Umbilical Cord Mesenchymal Stromal Cells for Cartilage Regeneration Applications. Stem Cells Int..

[12] Ashley J.R., Collier A.M., Olson S.D., Cox C.S. (2026). Freeze-Dried, Not Frozen: Lyophilized Mesenchymal Stromal Cell-Derived Extracellular Vesicles and Therapeutic Function in Neuroinflammatory Models. J. Am. Coll. Surg..

[13] Rajendran R.L., Onkar A., Batabyal R., Jha S.K., Mahajan A.A., Koley M., Banerjee S., P A., Saha M., Ghosh S. (2026). Stem Cell-Derived Extracellular Vesicles and Artificial Nanovesicles: A Translational Framework for Cell-Free Wound Repair. npj Regen. Med..

[14] Liu X., Wei Q., Lu L., Cui S., Ma K., Zhang W., Ma F., Li H., Fu X., Zhang C. (2023). Immunomodulatory Potential of Mesenchymal Stem Cell-Derived Extracellular Vesicles: Targeting Immune Cells. Front. Immunol..

[15] Wu T., Liu Y., Wang S., Shi C. (2025). MSC-Derived Extracellular Vesicles: Roles and Molecular Mechanisms for Tissue Repair. Int. J. Nanomed..

[16] Klyachko N.L., Arzt C.J., Li S.M., Gololobova O.A., Batrakova E.V. (2020). Extracellular Vesicle-Based Therapeutics: Preclinical and Clinical Investigations. Pharmaceutics.

[17] Luisotti L., Germelli L., Piccarducci R., Giacomelli C., Marchetti L., Martini C. (2025). Extracellular Vesicles as Vehicles for Small Non-Coding RNA Therapeutics: Standardization Challenges for Clinical Translation. Extracell. Vesicles Circ. Nucleic Acids.

[18] Zhao H., Li Z., Wang Y., Zhou K., Li H., Bi S., Wang Y., Wu W., Huang Y., Peng B. (2023). Bioengineered MSC-Derived Exosomes in Skin Wound Repair and Regeneration. Front. Cell Dev. Biol..

[19] Allan D., Tieu A., Lalu M., Burger D. (2020). Mesenchymal Stromal Cell-Derived Extracellular Vesicles for Regenerative Therapy and Immune Modulation: Progress and Challenges toward Clinical Application. Stem Cells Transl. Med..

[20] Kong L., Zhao G., Wu X., Ma S. (2026). Extracellular Vesicles in Cancer Diagnosis and Therapy: Advances, Challenges, and Prospects for Clinical Translation. Int. J. Mol. Sci..

[21] Li Q., Li Y., Shao J., Sun J., Hu L., Yun X., Liuqing C., Gong L., Wu S. (2025). Exploring Regulatory Frameworks for Exosome Therapy: Insights and Perspectives. Health Care Sci..

[22] Jia Y., Yu L., Ma T., Xu W., Qian H., Sun Y., Shi H. (2022). Small Extracellular Vesicles Isolation and Separation: Current Techniques, Pending Questions and Clinical Applications. Theranostics.

[23] Kim T., Hong J.W., Lee L.P. (2025). Efficient Methods of Isolation and Purification of Extracellular Vesicles. Nano Converg..

[24] Werle S.J., Therkelsen M.L.N., Meng C., Grønborg M., Gluud L.L., Damgaard D. (2026). No One-Size-Fits-All: An Evidence-Based Framework to Select Plasma EV Isolation Methods. bioRxiv.

[25] Théry C., Witwer K.W., Aikawa E., Alcaraz M.J., Anderson J.D., Andriantsitohaina R., Antoniou A., Arab T., Archer F., Atkin-Smith G.K. (2018). Minimal Information for Studies of Extracellular Vesicles 2018 (MISEV2018): A Position Statement of the International Society for Extracellular Vesicles and Update of the MISEV2014 Guidelines. J. Extracell. Vesicles.

[26] Welsh J.A., Goberdhan D.C.I., O’Driscoll L., Buzas E.I., Blenkiron C., Bussolati B., Cai H., Di Vizio D., Driedonks T.A.P., Erdbrügger U. (2024). Minimal Information for Studies of Extracellular Vesicles (MISEV2023): From Basic to Advanced Approaches. J. Extracell. Vesicles.

[27] Karimi N., Cvjetkovic A., Jang S.C., Crescitelli R., Hosseinpour Feizi M.A., Nieuwland R., Lötvall J., Lässer C. (2018). Detailed Analysis of the Plasma Extracellular Vesicle Proteome after Separation from Lipoproteins. Cell. Mol. Life Sci..

[28] Takakura Y., Hanayama R., Akiyoshi K., Futaki S., Hida K., Ichiki T., Ishii-Watabe A., Kuroda M., Maki K., Miura Y. (2024). Quality and Safety Considerations for Therapeutic Products Based on Extracellular Vesicles. Pharm. Res..

[29] Kawai-Harada Y., Nimmagadda V., Harada M. (2024). Scalable Isolation of Surface-Engineered Extracellular Vesicles and Separation of Free Proteins via Tangential Flow Filtration and Size Exclusion Chromatography (TFF-SEC). BMC Methods.

[30] Herrmann I.K., Wood M.J.A., Fuhrmann G. (2021). Extracellular Vesicles as a Next-Generation Drug Delivery Platform. Nat. Nanotechnol..

[31] Mohak S., Fabian Z. (2025). Extracellular Vesicles as Precision Delivery Systems for Biopharmaceuticals: Innovations, Challenges, and Therapeutic Potential. Pharmaceutics.

[32] Liam-Or R., Faruqu F.N., Walters A., Han S., Xu L., Wang J.T.-W., Oberlaender J., Sanchez-Fueyo A., Lombardi G., Dazzi F. (2024). Cellular Uptake and in Vivo Distribution of Mesenchymal-Stem-Cell-Derived Extracellular Vesicles Are Protein Corona Dependent. Nat. Nanotechnol..

[33] Phinney D.G., Pittenger M.F. (2017). Concise Review: MSC-Derived Exosomes for Cell-Free Therapy. Stem Cells.

[34] Adlerz K., Patel D., Rowley J., Ng K., Ahsan T. (2020). Strategies for Scalable Manufacturing and Translation of MSC-Derived Extracellular Vesicles. Stem Cell Res..

[35] Kim H.I., Park J., Zhu Y., Wang X., Han Y., Zhang D. (2024). Recent Advances in Extracellular Vesicles for Therapeutic Cargo Delivery. Exp. Mol. Med..

[36] Kaiser J.M., Eng T., Chihab S., Bowles-Welch A.C., Kippner L.E., Roy K., García A.J., Drissi H. (2026). Bone Marrow-Derived Mesenchymal Stromal Cells Yield Greater Pain Relief and Tissue Protection than Umbilical Cord Tissue-Derived Cells in a Surgically Induced Instability Model of Osteoarthritis. Osteoarthr. Cartil..

[37] Peng L., Jia Z., Yin X., Zhang X., Liu Y., Chen P., Ma K., Zhou C. (2008). Comparative Analysis of Mesenchymal Stem Cells from Bone Marrow, Cartilage, and Adipose Tissue. Stem Cells Dev..

[38] Mennan C., Brown S., McCarthy H., Mavrogonatou E., Kletsas D., Garcia J., Balain B., Richardson J., Roberts S. (2016). Mesenchymal Stromal Cells Derived from Whole Human Umbilical Cord Exhibit Similar Properties to Those Derived from Wharton’s Jelly and Bone Marrow. FEBS Open Bio.

[39] Thangavelu L., Mohan S., Alfaifi H.A., Farasani A., Menon S.V., Bansal P., Choudhary C., Kumar M.R., Vashishth R., Al-Rihaymee A.M.A. (2024). Safety and Efficacy of Stem Cell Therapy for Crohn’s Disease: An Umbrella Review of Systematic Reviews. Int. J. Surg..

[40] Planat-Benard V., Varin A., Casteilla L. (2021). MSCs and Inflammatory Cells Crosstalk in Regenerative Medicine: Concerted Actions for Optimized Resolution Driven by Energy Metabolism. Front. Immunol..

[41] Muslem S., AlTurani M., Maqsood M.B., Qaseer M.A. (2025). Cardiac Repair and Clinical Outcomes of Stem Cell Therapy in Heart Failure: A Systematic Review and Meta-Analysis. Diseases.

[42] Phinney D.G. (2026). A Precision Product Framework for Context-Dependent Mechanisms of MSC Therapy. Cell Stem Cell.

[43] Williams M.E., Banche Niclot F., Rota S., Lim J., Machado J., de Azevedo R., Castillo K., Adebiyi S., Sreenivasan R., Kota D. (2025). Optimizing Mesenchymal Stem Cell Therapy: From Isolation to GMP-Compliant Expansion for Clinical Application. BMC Mol. Cell Biol..

[44] Patel J.C., Shukla M., Shukla M. (2025). From Bench to Bedside: Translating Mesenchymal Stem Cell Therapies through Preclinical and Clinical Evidence. Front. Bioeng. Biotechnol..

[45] Chattopadhyay S., Rajendran R.L., Chatterjee G., Reyaz D., Prakash K., Hong C.M., Ahn B.-C., ArulJothi K.N., Gangadaran P. (2025). Mesenchymal Stem Cell-Derived Exosomes: A Paradigm Shift in Clinical Therapeutics. Exp. Cell Res..

[46] Jimenez-Puerta G.J., Marchal J.A., López-Ruiz E., Gálvez-Martín P. (2020). Role of Mesenchymal Stromal Cells as Therapeutic Agents: Potential Mechanisms of Action and Implications in Their Clinical Use. J. Clin. Med..

[47] Mayeen N.F., Salma U., Abu Kasim N.H., Mahmoud O., Haque N. (2025). Hurdles to Overcome for Mesenchymal Stem Cell Translation from Bench to Bedside. World J. Stem Cells.

[48] Levy O., Kuai R., Siren E.M.J., Bhere D., Milton Y., Nissar N., De Biasio M., Heinelt M., Reeve B., Abdi R. (2020). Shattering Barriers toward Clinically Meaningful MSC Therapies. Sci. Adv..

[49] Bagno L.L., Salerno A.G., Balkan W., Hare J.M. (2022). Mechanism of Action of Mesenchymal Stem Cells (MSCs): Impact of Delivery Method. Expert Opin. Biol. Ther..

[50] da Silva R.G.L., Au L., Blasimme A. (2024). Organizational Aspects of Tissue Engineering Clinical Translation: Insights from a Qualitative Case Study. Transl. Med. Commun..

[51] Baby H.M., Zhang H., Selvadoss A., Pathrikar T.V., Bajpayee A.G. (2026). Rational Design of Extracellular Vesicles for Targeted Drug Delivery across Physiological Barriers. Nano Today.

[52] Jo H.Y., Kim M.K., Kim K.T., Choi C., Rhee W.J. (2025). Extracellular Vesicles for Macrophage Reprogramming: An Emerging Paradigm in Immunomodulatory Therapeutics. J. Biol. Eng..

